# The relationship between teacher commitment, teacher self-efficacy, and work-related quality of life among science teachers

**DOI:** 10.1371/journal.pone.0326994

**Published:** 2025-07-01

**Authors:** Omar T. Bataineh, Ahmad M. Mahasneh, Zohair H. Al-Zoubi, Ahmad Qablan, Ahmed M. Alkaabi

**Affiliations:** 1 Department of Educational Foundations and Administration, The Hashemite University, Zarqa, Jordan; 2 Department of Educational Psychology, The Hashemite University, Zarqa, Jordan; 3 Department of C&I, The Hashemite University, Jordan and United Arab Emirates University, United Arab Emirates; 4 Department of Foundations of Education, United Arab Emirates University, United Arab Emirates; Universiti Sains Malaysia, MALAYSIA

## Abstract

This study examines the validity and reliability of the Arabic versions of the Teacher Commitment Scale, Teacher Self-Efficacy Scale, and Work-Related Quality of Life Scale. It explores the relationships among these variables in Jordan. A total of 616 science teachers participated by completing the three scales. Exploratory factor analysis indicated that the Arabic version of the Teacher Commitment Scale comprised four factors explaining 62.49% of the variance; the Teacher Self-Efficacy Scale consisted of one factor accounting for 60.22% of the variance; and the Work-Related Quality of Life Scale included six factors explaining 74.14% of the variance. Results showed statistically significant relationships among teacher commitment, self-efficacy, and quality of life. Future research should explore additional variables influencing teacher commitment.

## Introduction and background

Professional development may be regarded as a continuous learning experience for a teacher that begins before setting foot in a classroom and continues until retirement. The distinct aspects of teaching science are crucial elements in a teacher’s practice, training, and instruction, and they involve the use of programs specifically designed to promote positive achievement outcomes. In the long run, the entire professional development process is determined by educational policy in the best interests of student learning.

Professional development is a method whereby teachers gain practical experience to aid them in honing their knowledge, skills, and approach and becoming more competent and proficient [1]. In general, teachers are required to be familiar with current trends in educational practice, including recent advances in technology, educational systems, and policy, as well as other developments relevant to the effective performance of their role. Recent years have seen a wide range of changes in both the teaching and learning of science concerning instruction methods, assessment and examination guidelines, and learning emphasis, among others [2].

Nevertheless, Bell [3] emphasized the need for professional development for science teachers to carefully integrate science content knowledge and science process skills. With secondary school science teachers in mind, they noted the significance of teachers and students sharing these new experiences. Luft and Hewson building upon Hewson’s previous work and focusing on secondary school teachers, published an updated review of teacher professional development programs in science, stating that further research focusing on the importance of collaboration and emphasizing the success of professional learning development is needed [4]. However, they also noted the importance of understanding the links between policy, professional development programs, teachers, and students.

According to Shaharabani and Tal, professional development aims to assist secondary-level science teachers in enhancing their ability to support student learning effectively [5]. This involves enabling them to improve and develop their knowledge, practical skills, concepts, and convictions. While their research focused on the significant impact of professional development on the implementation and use of innovative curricula by secondary science teachers, their conclusions aligned with [6]. The latter asserted that sustained and intensive professional development programs were more effective than short-term programs.

### Organizational commitment

The term “organizational commitment” arose from the search to find effective, efficiently run organizations where managers and employees felt a bond with or empathy toward the company they worked for and where the ethos or psychology was based on human relations. Many recent studies have shown researchers’ interest in this concept, where employees’ actions and attitudes revealed their feelings toward the organization and their occupation and whether they felt a bond and a sense of belonging—a strong interpersonal rapport rather than simply an impersonal part of the organization [7,8].

Since the 1970s, organizational commitment has steadily gained importance in organizational psychology and is now understood as an individual’s psychological attachment to the organization. Having been established as an essential variable of a successful organization, the value of the concept has steadily increased to the point where, according to Özkalp and Kirel, organizational commitment is the key variable for organizational success and a prime management concept [9].

Klein and Park described organizational commitment as a multidimensional construct of a psychological state describing an employee’s relationship with their organization; it can determine whether the employee stays or leaves the organization [10]. Meyer and Allen described it as the degree to which an individual is involved with and believes in a specific organization [11]. Nazir and Ul Islam defined organizational commitment as the point reached by an employee in adhering to the behaviour required by the organization [12], while Herrera and De Las Heras-Rosas saw it as the employee’s wish to work hard, adopt the tenets of the organization, succeed, and remain an integral part of the organization [13]. Crosswell defined organizational commitment as an elevated attachment to an organization [14].

### Meyer & Allen’s model for commitment

Meyer and Allen introduced a three-component model of commitment that provides a comprehensive framework for understanding why individuals choose to remain committed to an organization [11]. Developed in the early 1990s, this model identifies three distinct dimensions of organizational commitment—affective, continuance, and normative. Each dimension represents a different psychological state that influences an individual’s attachment to their organization. Affective commitment stems from emotional attachment; continuance commitment is based on the perceived costs of leaving, and normative commitment reflects a moral obligation to stay. Together, these components offer valuable insights into employee behavior and motivation, making the model particularly relevant in education, where understanding teacher commitment is critical for improving retention and performance.

Affective commitment refers to the employee’s identification, involvement, and emotional attachment toward a particular organization. Continuous commitment is the term used for the employee’s reckoning of the costs incurred in leaving the organization, and normative commitment is the obligation felt by an employee to remain with the organization [15].

Brown and Sargeant pointed out that organizational commitment was a significant feature of educational institutions; from the teachers’ perspective, it is the commitment to the school or other educational establishment—a combination of overt and covert contracts between the teachers and the school, which includes a psychological aspect [16]. These contracts illustrate the degree of normality and natural behaviour in the teacher-school relationship; when looked at from the point of view of value to society, this commitment does not breed opportunism. According to Erdem organizational commitment’s importance lies in establishing lasting and mutually beneficial relationships between teachers and their schools [17]. Balay reported greater teacher satisfaction for those working in successful schools than in less successful institutions [18].

Shapira-Lishchinsky and Rosenblatt found that teachers with low organizational commitment had scant regard for time-keeping and abused sick days [19], which reflected poorly on pupils’ learning through cancellation of classes or low-grade substitute teachers [20]. In addition, teachers with low organizational commitment tended to switch frequently from one school to another or to leave the teaching profession entirely [21]. Xaba pointed out that employing inexperienced replacement teachers lowered the school’s professional educational standards; less experienced, ineffective teachers contributed to diminished student achievement [22,23]. Fresko et al. found that rather than having the school’s best interests at heart, teachers with low organizational commitment were only interested in their success and gave little thought to the quality of education they provided [24]. The teachers’ priorities are subsequently reflected in the students’ academic achievement [25].

Shu described a committed teacher as hard-working and dedicated to fulfilling their commitment to the pupils and the school academically through strict time-keeping, undertaking extracurricular activities, encouraging and influencing student and school goals and achievements, extending their efforts beyond self-interest and ambition, and intending to remain at the school long-term [26]. Ibrahim et al. and Yukl commented that awareness and understanding of their level of commitment and involvement in the school were critical to assessing a teacher’s agreement with and support for school policy and objectives [27,28]. Anthonysamy defined motivational beliefs as being mindful of the significance of a task to be fulfilled or the acquisition of information or skill and having the ability, responsive inclination, and specific belief in one’s aptitude and capability to achieve it [29].

### Theoretical framework: Bandura’s social cognitive theory of self-efficacy

Albert Bandura’s Social Cognitive Theory emphasizes the role of observational learning, social experiences, and reciprocal interactions among behavior, personal factors, and the environment in shaping human behavior. A central element of this theory is self-efficacy—a person’s belief in their ability to succeed in specific situations or accomplish a task.

According to Bandura self-efficacy plays a key role in how goals are approached, how much effort is exerted, and how resilient individuals are in the face of challenges [30–32]. High self-efficacy enhances motivation, persistence, and achievement, while low self-efficacy can result in avoidance, stress, and poor performance.

Bandura identified four main sources of self-efficacy:

1. Mastery Experiences – Successes build a robust belief in one’s capabilities, while failures, especially early on, can undermine it.
2. Vicarious Experiences—Observing others perform successfully can strengthen one’s beliefs in one’s abilities, particularly when the observer sees the model as similar to themselves.
3. Verbal Persuasion—Encouraging feedback and affirmations from others can enhance self-belief, though they are generally less potent than experiential sources.
4. Physiological and Emotional States – Positive mood states support self-efficacy, while stress, anxiety, or fatigue can diminish it.

In educational settings, teacher self-efficacy impacts instructional quality, classroom management, student engagement, and openness to innovation. Teachers who believe in influencing student learning tend to demonstrate greater effort, persistence, and adaptability in their professional practice.

According to Bandura an individual’s life was molded more by their perceptions and beliefs than by objective realities; consequently, the belief in one’s capability to control one’s behavior and influence important happenings in one’s life gained added significance [31]. Doğan used it to explain the difference between an individual’s performance in a field and their potential—also known as self-efficacy [33]. Doménech-Betoret et al., maintained that self-efficacy beliefs applied to satisfactory task completion were more effective than skills [34]. Tschannen-Moran and Hoy argued that teachers were expected to believe strongly in self-efficacy [35], while Pajares and Zimmerman described self-efficacy as an individual’s belief in their ability to respond appropriately and successfully to life events [36–38]. Pajares added that self-efficacy was the combination of an individual’s direct experience, observation of social models (indirect experience), verbal judgments (from people in the environment), and levels of emotional readiness [36]. Bandura and Pajares also found that an individual’s cognitive, affective, motivational, and selection processes were affected by belief in self-efficacy [31,36]. Teachers’ belief in self-efficacy was positively or negatively affected by success or failure in their professional lives.

Observation of colleagues’ experience (indirect experience) in similar or familiar situations may impact teachers’ self-efficacy positively or negatively, depending on the colleague’s success or failure in any given situation. Teachers respond, as do people in general, to positive verbal support and appreciation, which increases their belief in their self-efficacy. Mental and emotional circumstances are important factors that provide the necessary affective competence to fulfill a task, and self-efficacy beliefs rely on factors such as teacher satisfaction that result from a positive attitude and enjoyment of teaching [30]. Teachers’ cognitive processes are reflected in various tasks, and the emotional processes involved in stress management, hard work, energy, risk-taking decisions, and internal motivation processes allow them to carry out their duties [38]. Soto and Rojas also stated that competent, high-performing teachers in these aspects were unlikely to avoid tasks, and they judged this to reflect their job satisfaction [39].

Bakar et al. and Sharma et al. reiterated the findings of previous studies that illustrated strong links between efficacy beliefs and teacher performance when it accompanied positive student results and a positive attitude toward teaching [40,41]; they are also strong indicators of successful future careers in teaching [42]. Tschannen-Moran et al. found that belief in self-efficacy began to develop early in teacher training and is task-specific [43].

Employees judge their work-related life quality by how relaxed and happy they feel at work [44]. Sirgy et al. defined quality of work-related life as how contented the employee felt regarding job position or grade, salary, professional growth, job security, balance between professional and private life, and good relationships with colleagues [45]. They also noted the importance of the working atmosphere, such as cheerfulness and subjective well-being. Lau and May included the importance of a supportive and encouraging working atmosphere in promoting employee satisfaction, stimulating employee interest and effort, and providing job security, a good salary, and adequate opportunities for professional development [46]. Royuela et al. included various dimensions of working life: the intrinsic value of work, skills development and career advancement, work organization, health and safety, stability, and a comfortable balance between work commitments and private, family, and social life [47].

Fang & Qi (2023) examine how school climate impacts teachers’ job satisfaction, focusing on the mediating role of self-efficacy [48]. The study finds that a favorable school climate leads to higher teacher self-efficacy, which, in turn, enhances job satisfaction. This suggests that creating a supportive and positive school environment can foster teachers’ confidence in their abilities, leading to greater job satisfaction and overall well-being.

Huang et al., explore the complex relationships between self-efficacy, job satisfaction, and occupational commitment [49]. They argue that self-efficacy is a crucial factor influencing job satisfaction, subsequently affecting teachers’ commitment to their profession. Their findings highlight that enhancing teachers’ self-belief can improve their job satisfaction and strengthen their commitment, which is essential for retaining teachers.

Ortan et al., examine the relationship between teacher self-efficacy, job satisfaction, and overall well-being within K-12 schools [50]. The study finds that higher self-efficacy positively correlates with increased job satisfaction and improved teacher well-being. They suggest that fostering self-efficacy in teachers is vital for enhancing their mental and emotional health, which, in turn, leads to more positive teaching experiences and better job satisfaction.

In the study Relations of Science Teaching Self-efficacy with Instructional Practices, Student Achievement, and Support, and Teacher Job Satisfaction, Perera et al., explore how self-efficacy in science teaching is linked to instructional practices, student achievement, and teacher job satisfaction [51]. Their findings show that teachers with higher science teaching self-efficacy are more likely to implement effective instructional strategies, positively impacting student achievement and teacher job satisfaction. This study emphasizes the importance of boosting self-efficacy in science teachers to enhance both teaching quality and personal job satisfaction.

Lastly, the comparative study by Guoyan et al., investigates how self-efficacy, mental well-being and commitment to using learning management systems (LMS) influenced teachers during the COVID-19 pandemic [52]. The study found that higher self-efficacy and better mental well-being were associated with greater commitment to using LMS platforms, even amidst the challenges of the pandemic. This research highlights the critical role of self-efficacy and mental health in sustaining teacher engagement with technology and online teaching during a crisis.

These studies underscore teacher self-efficacy’s significant role in shaping various aspects of their professional lives. Whether it is job satisfaction, occupational commitment, well-being, or instructional practices, strengthening teachers’ self-belief is a key factor in enhancing overall teacher performance and satisfaction. These studies suggest that fostering self-efficacy is essential, particularly in challenging circumstances such as the COVID-19 pandemic, to support teachers and improve their engagement with teaching and professional responsibilities.

### Research problem

Teachers are not just educators but pivotal Figs in shaping students’ educational and social development. They carry immense responsibilities for imparting knowledge, fostering a positive school environment, supporting their colleagues, and engaging in continuous professional development. However, despite teachers’ essential role, the factors influencing their work commitment are complex and multifaceted. Recent research has underscored the importance of communicative strategies [53] and intercultural dynamics [54] in shaping teachers’ commitment. These studies highlight that internal, personal factors do not solely drive teacher commitment but are also influenced by broader sociocultural contexts. Understanding teacher commitment through a sociocultural lens is crucial for understanding how it is shaped.

However, despite the recognized importance of teacher commitment, there remains a significant gap in the existing literature. While much attention has been given to various aspects of teacher motivation and professional engagement, little research has explored the relationship between teacher commitment, teacher self-efficacy, and work-related quality of life—especially within the context of science teachers. This gap is particularly glaring in the case of Jordan, where no qualitative or quantitative studies have investigated how these key factors interconnect in the lives of teachers.

Addressing this gap is not just an academic exercise—it has practical implications for improving teacher well-being, job satisfaction, and student outcomes. The current study aims to fill this critical gap by focusing on science teachers in Jordan. It seeks to examine the validity and reliability of the Arabic versions of established scales—such as the Teacher Commitment Scale, Teacher Self-Efficacy Scale, and Work-Related Quality of Life Scale—and to investigate the relationships among these variables. The study will shed light on how teacher commitment, self-efficacy, and work-related quality of life are interrelated, offering valuable insights for educators and policymakers.

The Teacher Commitment Scale (TCS), Teacher Self-Efficacy Scale (TSES), and Work-Related Quality of Life Scale (WRQoL) were all used in the study, though the specific rationale for their selection was not explained. These scales are widely used in educational research to measure teacher engagement, confidence, and well-being. To understand the choice of these tools. It is crucial to explore how they are applied in relevant research fields and why they were likely selected for this study.

TCS measures a teacher’s dedication to their profession, including emotional attachment, professional identity, and commitment to student success. This scale is commonly used in studies examining teacher retention, job satisfaction, and performance. It is well-established that teacher commitment plays a significant role in job satisfaction and teacher effectiveness [20,24]. In this study, the TCS was likely chosen to assess how teachers perceive their commitment to the profession and how this relates to other variables like self-efficacy and job satisfaction. By measuring teacher commitment, the researchers could better understand how emotional attachment and professional dedication influence teachers’ job satisfaction and overall professional experience.

The Teacher Self-Efficacy Scale (TSES) assesses teachers’ belief in managing classroom challenges, implementing effective teaching strategies, and promoting student success. Self-efficacy is closely related to teacher motivation, job satisfaction, and student achievement [35]. The TSES was a logical choice in this study because it measures teachers’ self-perception regarding their teaching capabilities. Given the study’s focus on teacher self-efficacy and job satisfaction, the TSES offered a reliable means to quantify teachers’ confidence in their professional abilities. This scale enabled the researchers to examine how teachers’ belief in their effectiveness impacts their satisfaction and engagement with their work.

The Work-Related Quality of Life Scale (WRQoL) evaluates different dimensions of an individual’s work-related well-being, including job satisfaction, work-life balance, and stress. This scale has been widely used in organizational psychology to understand how work environments affect employees’ quality of life. The WRQoL is especially valuable for teachers because it captures factors like job-related stress, burnout, and satisfaction with workplace conditions. In this study, the WRQoL was likely chosen to assess the broader context of teachers’ professional experiences, looking at how their work environment and emotional well-being contribute to their overall job satisfaction. Measuring work-related quality of life helped the researchers understand how different aspects of the teaching profession, such as stress and work-life balance, influence teachers’ overall satisfaction and commitment.

These scales provide a multidimensional approach to studying teacher well-being, commitment, and self-efficacy. They are widely validated and have been shown to reliably assess factors influencing job satisfaction, teacher motivation, and professional commitment. Their use in this study allowed the researchers to explore how teacher self-efficacy, commitment, and work-related well-being interact and contribute to job satisfaction. The scales offer a robust framework for investigating these relationships and provide valuable insights into improving teacher engagement, satisfaction, and retention.

## Methodology

### Study design

This study adopted the descriptive-correlational approach because it is appropriate for achieving its objectives. Means and standard deviations were calculated for teacher commitment, self-efficacy, and work-related quality of life. The Pearson correlation coefficient was used to investigate the relationship between these factors. SPSS version 28 was used to perform the needed statistical analysis.

### Sample

All teachers from Jerash Governorate were invited to participate in this study. However, only 616 science teachers responded to the questionnaires. The returned questionnaires had the following demographic characteristics: 62.5% had bachelor’s degrees, and 37.5% had postgraduate qualifications. Participants had varying years of teaching experience: 33% had 1–5 years, 40% had 6–10 years, 10% had 11–15 years, 12% had 16–20 years, and 5% had more than 21 years of teaching experience. The teachers’ ages ranged between 23 and 55 years.

The sample of 616 science teachers is statistically sufficient for the study, offering a robust and reliable data set. The sample size exceeds the recommended threshold for psychometric analyses, ensuring strong validity for the scales used. Additionally, the demographic diversity in educational qualifications, teaching experience (ranging from 1 to over 21 years), and age (23–55 years) enhances the sample’s representativeness. Despite not all invited teachers responding, the large number of participants provides a comprehensive view of the teacher population in the governorate, making the data reliable and relevant to the study’s objectives.

### Instruments

This study used three scales: The Teacher Commitment Scale, the Teacher Self-Efficacy Scale, and the Work-Related Quality of Life Scale. Below is a description of these scales in their original form.

### Teacher commitment scale (TCS)

TCS was developed by Thien et al., [55]. It consists of 13 items distributed over four dimensions: commitment to school (3 items, α = 0.89), commitment to students (3 items, α = 0.82), commitment to teaching (3 items, α = 0.73), and commitment to the profession (4 items, α = 0.71). Responses are graded on a 6-point Likert scale ranging from 1 (*Strongly Disagree*) to 6 (*Strongly Agree*).

### Teacher self-efficacy scale (TSES)

The Teacher Self-Efficacy Scale (TSES), developed by Schwarzer et al., comprises 10 items [56]. In the current study, internal consistency was measured using Cronbach’s alpha, yielding a reliability coefficient of α = 0.82, which is comparable to the reliability reported by Schwarzer et al., [56]. Responses to TSES items were rated on a 4-point Likert scale ranging from 1 (*Not at All True*) to 4 (*Exactly True*).

### Work-related quality of life scale (WRQoLS)

WRQoLS was developed by Easton and Laar and consists of 23 items distributed over six dimensions [57]: general well-being (6 items, α = 0.88), homework interface (3 items, α = 0.82), job–career satisfaction (3 items, α = 0.86), control at work (4 items, α = 0.81), work conditions (4 items, α = 0.75), and stress at work (2 items, α = 0.81). The internal consistency using Cronbach’s alpha for overall WRQoLS was α = 0.91; responses to WRQoLS items were rated on a 5-point Likert scale ranging from 1 (*Strongly Disagree*) to 5 (*Strongly Agree*). Before data collection, The Arabic versions of the scales underwent forward-backward translation, aligned with best practices for intercultural research [54]. Additionally, interviews with 20 science teachers were held to ensure item clarity and mirroring procedures used by Rasool et al., Thien et al., and Easton & Laar for feedback validity [55,57,58].

### Data collection and analysis

Following the translation of the study scales from English to Arabic and the validation of translation accuracy. After getting ethical approval from the Hashemite University IRB (No.35/7/2022/2023), researchers distributed the study tools to participants, visited the schools, met the teachers, and clarified the purpose of the study. Participants were informed about the nature of the study. Their written consent forms were obtained before they were sent to respond to the three questionnaires. Data collection spanned two months, from November 1, 2023, until December 30, 2023. After this, the data were entered and stored electronically. Data analysis was conducted using SPSS Version 28. Exploratory factor analysis using principal components analysis was performed to check the validity of the Arabic versions of the Teacher Commitment Scale, Teacher Self-Efficacy Scale, and Work-Related Quality of Life Scale. Internal consistency was assessed using Cronbach’s alpha to check the reliability of the three scales. Means and standard deviations were calculated for teacher commitment, self-efficacy, and work-related quality of life. The Pearson correlation coefficient was used to investigate the relationship between teacher commitment, teacher self-efficacy, and work-related quality of life.

## Results

### Results of objective one

#### Exploratory factor analysis for TCS.

Exploratory factor analysis using principal components analysis was conducted to assess the validity of the TCS Arabic version. The Kaiser-Meyer-Olkin (KMO) measure of sampling adequacy was 0.709, and Bartlett’s test of sphericity was significant, χ²(78) = 2,412.882, p < .001. Table 1 presents the factor loadings and the total variance explained.

**Table 1:** Factors Loadings of the Teacher Commitment Scale.

Items	School	Student	Teaching	Profession
**1**	0.64			
**2**	0.80			
**3**	0.57			
**4**		0.44		
**5**		0.59		
**6**		0.84		
**7**			0.63	
**8**			0.67	
**9**			0.66	
**10**				0.65
**11**				0.75
**12**				0.76
**13**				0.77
**Eigenvalue**	2.688	2.163	1.918	1.356
**% of Variance**	20. 680	16.636	14.750	10.430
**Cumulative %**	20.680	37.316	52.066	62.496

**Fig 1:**
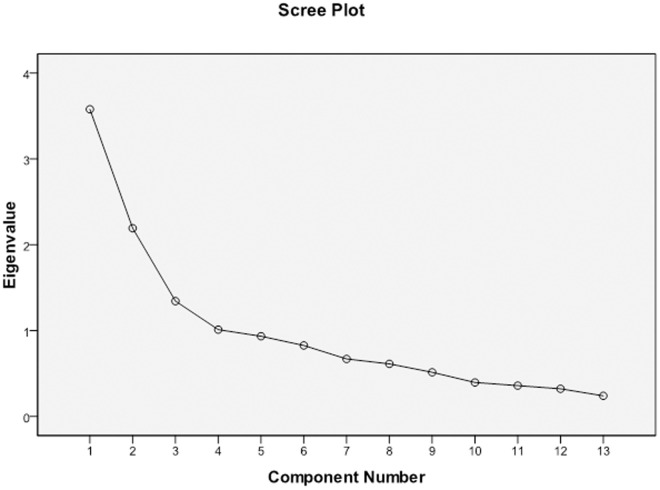
Scree Plot for the TCS Arabic Version.

Table 1 indicates four factors with eigenvalues greater than 1, which explains 62.49% of the variance. Fig 1 shows the scree plot of the TCS Arabic version.

As shown in Fig 1, the eigenvalues decline sharply across the first four components before the curve begins to level off from the fifth component onward. According to Cattell’s scree test [59], the point of inflection—often referred to as the “elbow”—appears at Component 4, suggesting that a four-factor solution is the most appropriate representation of the underlying structure of the TCS. This finding differs from the six-factor model proposed by Thien et al., possibly due to cultural or contextual differences in the sample [55]. The four retained factors capture the majority of the meaningful variance in the data, supporting their theoretical and empirical relevance in the current study.

Internal consistency of the TCS Arabic version was assessed using Cronbach’s alpha, which was 0.79 overall and 0.81, 0.80, 0.71, and 0.75 for commitment to school, commitment to students, commitment to teaching, and commitment to the profession, respectively.

#### Exploratory factor analysis for TSES.

Exploratory factor analysis using principal components analysis was conducted to assess the validity of the TSES Arabic version. The KMO measure was 0.866, and Bartlett’s test of sphericity was significant, χ²(45) = 3,024.540, p < .001. Table 2 presents the factor loadings, eigenvalues, and the total variance explained.

**Table 2:** Factors Loadings of the Teacher Self-Efficacy Scale.

Items	Teacher Self-Efficacy
**1**	0.68
**2**	0.41
**3**	0.64
**4**	0.65
**5**	0.60
**6**	0.85
**7**	0.75
**8**	0.86
**9**	0.84
**10**	0.75
**Eigenvalue**	6.023
**% of Variance**	60.22
**Cumulative %**	60.22

One factor with an eigenvalue greater than 1 accounted for 62.22% of the variance. It is worth mentioning that item 2’s loading (0.41) has a moderate loading and is considered acceptable, especially in exploratory factor analysis (EFA) [60]. It indicates that the factor explains about 16.8% of the variance in that item.

**Fig 2:**
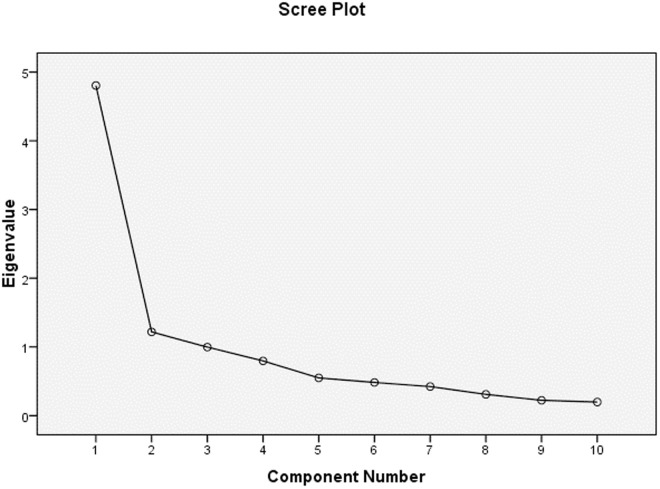
Scree Plot for the TSES Arabic Version.

Fig 2 shows the scree plot of the TSES Arabic version. Cronbach’s alpha for the TSES was 0.85, which indicated good internal consistency.

#### Exploratory factor analysis for WRQoLS.

Exploratory factor analysis using principal components analysis was conducted to assess the validity of the WRQoLS Arabic version. The KMO measure was 0.884, and Bartlett’s test of sphericity was significant, χ²(253) = 10,655, p < .001. Table 3 presents the factor loadings, eigenvalues, and the total variance explained.

**Table 3:** Factors Loadings of the Work-Related Quality of Life Scale.

Items	GWB	HWI	JCS	CAW	WC	SAW
**4**	0.54					
**9**	0.49					
**10**	0.75					
**15**	0.54					
**17**	0.83					
**21**	0.68					
**5**		0.48				
**6**		0.87				
**14**		0.54				
**1**			0.72			
**3**			0.64			
**8**			0.58			
**11**			0.87			
**18**			0.69			
**20**			0.68			
**2**				0.60		
**12**				0.65		
**23**				0.60		
**13**					0.48	
**16**					0.67	
**22**					0.77	
**7**						0.67
**19**						0.83
**Eigenvalue**	4.112	3.656	3.282	2.866	1.602	1.535
**% of Variance**	17.877	15.896	14.269	12.461	6.966	6.672
**Cumulative %**	17.877	33.773	48.041	60.502	67.468	74.140

*Note.* GWB = General Well-Being; HWI = Home–Work Interface; JCS = Job–Career Satisfaction; CAW = Control at Work; WC = Work Conditions; SAW = Stress at Work.

**Fig 3:**
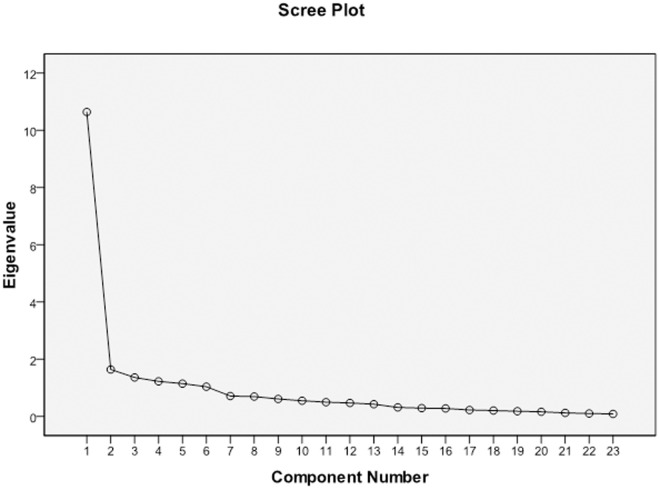
Scree Plot for the WRQoLS Arabic Version.

Six factors had eigenvalues greater than 1, which captured 74.14% of the variance. Fig 3 shows the scree plot of the WRQoLS Arabic version.

Internal consistency was assessed using Cronbach’s alpha, which was 0.87 overall, and 0.80, 0.78, 0.84, 0.75, 0.73, and 0.86 for general well-being, homework interface, job–career satisfaction, control at work, working conditions, and stress at work, respectively.

### Results of objective two

Table 4 presents the descriptive statistics of the study variables, including means, standard deviations, skewness, kurtosis, and minimum and maximum scores. For the teacher commitment dimensions measured on a 6-point Likert scale, the mean scores ranged from 3.33 to 3.59.

**Table 4:** Descriptive Statistics of Teacher Commitment, Teacher Self-Efficacy, and Quality of Working Life.

Variables	Mean	SD	Skewness	Kurtosis	Min	Max
**Commitment to School**	3.59	0.57	−0.012	0.033	1	6
**Commitment to Students**	3.56	0.88	−0.295	−0.409	1	6
**Commitment to Teaching**	3.43	0.63	0.061	−0.506	1	6
**Commitment to Profession**	3.33	0.91	0.031	−0.899	1	6
**Teacher Commitment**	3.47	0.53	0.183	−0.604	1	6
**Teacher Self-Efficacy**	3.35	0.72	−0.077	−0.253	1	4
**General Well-Being**	3.17	0.80	0.014	−0.200	1	5
**Homework Interface**	3.41	0.82	−0.338	0.322	1	5
**Job and Career Satisfaction**	3.17	0.85	−0.213	−0.354	1	5
**Control at Work**	3.04	1.04	0.130	−0.770	1	5
**Working Conditions**	3.31	0.84	0.115	−0.570	1	5
**Stress at Work**	2.94	0.99	0.185	−0.666	1	5
**Work-Related Quality of Life**	3.17	0.77	0.095	−0.386	1	5

Based on the skewness and kurtosis values shown in Table 4, the data for the variables generally appear to meet the assumption of normality required for parametric statistical methods. Specifically, Skewness values are within the acceptable range (−0.5 to 0.5), indicating minimal distribution asymmetry. Kurtosis values also fall within an acceptable range, suggesting that the distributions are neither too flat nor too peaked and are close to the shape of a normal distribution.

Table 4 shows that the level of teacher commitment was moderate (M = 3.35), teacher self-efficacy was high (M = 3.35), and teacher work-related quality of life was moderate (M = 3.17). These Figs seem to be normal considering the high teaching load of Jordanian teachers, which resulted from the massive number of Syrian and other refugees who fled to the country and overwhelmed its school system. Pearson correlation coefficients were calculated to investigate the relationships between these variables, as shown in Table 5.

**Table 5:** Pearson Correlation Matrix Between Study Variables.

Variables	Teacher Commitment	Teacher Self-Efficacy	Work-Related Quality of Life
**Teacher Commitment**	1		
**Teacher Self-Efficacy**	0.56*	1	
**Work-Related Quality of Life**	0.53*	0.76*	1

*Note.* *p* = .01.

Table 5 shows statistically significant correlations between teacher commitment and teacher self-efficacy (r = .56, p < .001), between teacher commitment and work-related quality of life (r = .53, p < .001), and between teacher self-efficacy and work-related quality of life (r = .76, p < .001).

The results of the multiple regression analysis shown in Table 6 indicate that self-efficacy and work-related quality of life significantly influenced teacher commitment. The R² value was.314 for self-efficacy, which indicated that self-efficacy accounted for 31.4% of the variance in teachers’ commitment. The R² value was.290 for work-related quality of life, which captured 29% of the variance in commitment. The F values from the ANOVA were F(1, 614) = 281.072, p < .001 for self-efficacy, and F(1, 614) = 250.571, p < .001 for work-related quality of life.

**Table 6:** Multiple Regression Results: Teacher Self-Efficacy and Work-Related Quality of Life as Predictors of Teacher Commitment.

Variables		R	R^2^	F	β	T	Sig	CI
**Teacher Self-Efficacy**	**Teacher Commitment**	0.560	0.314	281.072	0.560	16.765	0.00	[0.494, 0.625]
**Work-Related Quality of Life**		0.538	0.290	250.571	0.538	15.831	0.00	[0.471, 0.604]

The results indicate that Teacher Self-Efficacy and Work-Related Quality of Life are moderately strong predictors of Teacher Commitment. Specifically, Teacher Self-Efficacy shows an R value of 0.560 and explains approximately 31.4% (R^2^ = 0.314) of the variance in Teacher Commitment. The corresponding F-value (281.072) and highly significant p-value (p = 0.00) suggest a statistically robust relationship. The standardized beta coefficient (β = 0.560) and high t-value (t = 16.765) further emphasize the strength and significance of Teacher Self-Efficacy as a predictor.

Similarly, Work-Related Quality of Life presents an R-value of 0.538, with an R^2^ of 0.290, indicating that it explains 29.0% of the variance in Teacher Commitment. Again, the high F-value (250.571) and significant p-value (p = 0.00) confirm the reliability of this relationship. The standardized beta coefficient (β = 0.538) and t-value (t = 15.831) show that although it is slightly weaker than Teacher Self-Efficacy, Work-Related Quality of Life remains a meaningful predictor.

Both predictors demonstrate moderate predictive strength and statistically significant relationships with Teacher Commitment. However, a considerable proportion of the variance (around 69–71%) remains unexplained, suggesting that additional factors, possibly including leadership support, work environment, and personal factors, might also play a critical role. Therefore, it would be crucial to investigate further the extent to which school leadership practices and workload contribute to the explained variance. Leadership factors might exert a more strategic or motivational influence, while workload may stress commitment or performance. Disentangling their specific impacts would provide deeper insights into how organizational conditions shape individual outcomes.

## Discussion

This study aimed to assess the validity and reliability of the teacher commitment, self-efficacy, and work-related quality of life scales among a sample of Jordanian science teachers. The results of exploratory factor analysis showed that four factors of the TCS Arabic version explained 62.49% of the total variance, which supported internal consistency. Thien et al., noted that their exploratory factor analysis revealed six factors of the Teacher Commitment Scale [55]. The exploratory factor analysis results indicated that one factor of the TSES Arabic version explained 60.22% of the total variance and thus confirmed internal consistency. Although Thien et al., suggested a six-factor structure, TCS appears to exhibit a four-factor solution in this context [55]. This discrepancy may be attributed to potential cultural differences, and it would be valuable in future studies to explore these cultural factors to understand the variation in the factor structure.

Schwarzer et al., reported that their exploratory factor analysis showed one factor in the Teacher Self-Efficacy Scale [56]. Similarly, the exploratory factor analysis demonstrated that six factors of the WRQoLS Arabic version explained 74.14% of the total variance. Easton and Laar found that their exploratory factor analysis identified six factors in the Work-Related Quality of Life Scale [57].

The results showed a positive correlation between teachers’ commitment and their self-efficacy. The researchers attribute this finding to the positive effect of commitment on individuals’ self-belief, psychological well-being, and personal health. Commitment plays an important role in teacher competence, providing a sense of security, ability, adequacy, and self-confidence, thereby ensuring psychological stability. Through social interaction, teachers perform their professional roles, satisfy their needs, fulfill their desires, and express themselves, simultaneously reducing tension and anxiety. Individuals’ self-concept motivates them to strive toward self-achievement through effort and excellence. Teachers who are content with themselves and their roles are likelier to work with dedication, show perseverance, and strive for excellence. Likewise, teachers who set goals and levels of ambition based on a realistic appraisal of their potential will achieve success, leading to self-acceptance and increasing engagement in work and achievement. Thus, a high level of teacher commitment positively impacts self-efficacy.

The significant correlations observed in the study between teacher commitment and work-related quality of life (r = .53, p < .001) and between teacher self-efficacy and work-related quality of life (r = .76, p < .001) indicate strong positive relationships among the variables examined. These findings are consistent with previous research conducted among Jordanian teachers, which has documented similar associations between job satisfaction, motivation, leadership behaviors, and organizational commitment [48]. For instance, a study by Khasawneh et al. found a substantial positive correlation (r = 0.50, *p* < 0.001) between principals’ transformational leadership behaviors and vocational teachers’ organizational commitment in Jordan [61]. This suggests that leadership styles significantly influence teachers’ commitment levels. Additionally, research by Abu Shaama demonstrated significant positive correlations between job satisfaction, work engagement, and organizational commitment among employees in Jordanian telecommunication companies, with correlation coefficients of r = 0.719 and r = 0.795, respectively [62]. Although this study was conducted in a different sector, the findings underscore the critical role of job satisfaction and engagement in fostering organizational commitment.

Furthermore, a study focusing on public school teachers in Mafraq Province, Jordan, reported a statistically significant relationship between motivation and job satisfaction, with a correlation coefficient of r = 0.11 (*p* = 0.02). While this correlation is modest, it highlights the interconnectedness of motivational factors and job satisfaction among Jordanian teachers. These studies corroborate the findings, emphasizing the importance of leadership behaviors, job satisfaction, and motivation in enhancing organizational commitment among teachers in Jordan.

The correlation matrix revealed moderate positive relationships between Teacher Commitment and two key variables: Teacher Self-Efficacy (r = 0.560) and Work-Related Quality of Life (r = 0.538). The positive direction of these relationships suggests that as teachers’ sense of self-efficacy and their perceptions of work-related quality of life improve, their commitment to the profession also increases. The moderate strength of these correlations indicates that while these variables are significant, other factors are likely influencing Teacher Commitment as well [63].

The findings align with established theories. Bandura’s Social Cognitive Theory emphasizes the role of self-efficacy in shaping motivation and perseverance, helping explain why teachers who believe in their capabilities tend to stay committed [31]. Similarly, Herzberg’s Two-Factor Theory highlights the importance of positive work conditions in fostering job satisfaction and organizational commitment [64]. Thus, teachers experiencing a higher quality of work life are more likely to feel connected and loyal to their profession.

These results have important implications for future research and practice. Future studies should investigate additional influences on Teacher Commitment, such as leadership practices, emotional exhaustion, and school culture. For practice, interventions aiming to boost teacher self-efficacy [31] and enhance the quality of work environments [64] could be effective strategies for increasing teacher retention and professional commitment.

The correlation matrix revealed moderate positive relationships between Teacher Commitment and two key variables: Teacher Self-Efficacy (r = 0.560) and Work-Related Quality of Life (r = 0.538). The positive direction of these relationships suggests that as teachers’ sense of self-efficacy and perceptions of work-related quality of life improve, their commitment to the profession also increases. The moderate strength of these correlations indicates that while these variables are significant, other factors likely influence Teacher Commitment [63].

The findings align with established theories. Bandura’s Social Cognitive Theory emphasizes the role of self-efficacy in shaping motivation and perseverance, helping explain why teachers who believe in their capabilities tend to stay committed [31]. Similarly, recent research [65] highlights the critical role of work-related quality of life in influencing employee satisfaction, well-being, and organizational commitment. Thus, teachers experiencing a higher quality of work are more likely to feel connected and loyal to their profession.

These results have important implications for future research and practice. Future studies should investigate additional influences on Teacher Commitment, such as leadership practices, emotional exhaustion, and school culture. For practice, interventions aiming to boost teacher self-efficacy [31] and enhance the quality of work environments [65] could be effective strategies for increasing teacher retention and professional commitment.

The regression analysis also revealed that Teacher Self-Efficacy and WRQoL are significant predictors of Teacher Commitment. Specifically, Teacher Self-Efficacy explained 31.4% of the variance in commitment scores (R^2^ = 0.314, F = 281.072, β = 0.560, *p* < .001), while WRQoL accounted for 29.0% of the variance (R^2^ = 0.290, F = 250.571, β = 0.538, *p* < .001). These R^2^ values indicate moderate predictive strength, suggesting that each variable contributes meaningfully to understanding teacher commitment.

The slightly higher predictive power of self-efficacy aligns with Bandura’s social cognitive theory [31], which posits that belief in one’s teaching competence is central to sustained motivation and perseverance in the face of challenges. Meanwhile, the strong influence of WRQoL reflects the impact of the workplace environment and perceived support on teachers’ emotional attachment and sense of belonging within the school context.

Differences in variance may also reflect institutional and contextual factors such as school leadership styles and workload distribution. For instance, supportive leadership has been linked to enhanced teacher efficacy and better perceptions of workplace quality [66], while excessive workload is often associated with burnout and reduced commitment [67]. Therefore, these findings underscore the need for school systems to consider organizational dynamics when fostering teacher engagement and retention.

### Teachers’ perceptions, self-efficacy *&* efforts

According to teachers’ perceptions of self-efficacy, their educational efforts, enthusiasm, and objectives vary. Moreover, each factor influences their thinking patterns, problem-solving skills, and emotional reactions. Teachers with low self-efficacy tend to be negative, have poor problem-solving skills, and perceive problems as more complex than they are, whereas teachers with high levels of self-efficacy are confident, have firm resolve, and possess better problem-solving skills [43,68–70]. When dealing with problems and difficult situations in their professional lives, teachers with high self-efficacy shoulder responsibility with determination and confidence in their success. In so doing, they expand their organizational commitment and improve their work-related quality of life.

The results indicated a positive correlation between teachers’ commitment and their work-related quality of life. The researchers attributed this finding to the fact that the quality of work-related life contributes to the development and strengthening of professional commitment among teachers and enhances it by providing the resources, tools, and competencies to test and implement their ideas. Transformational leadership that provides the resources teachers need and involves them in decision-making strengthens teachers’ commitment at a high level.

### Situating the findings of this study within the international context

Shahrashoob conducted a study among Iranian secondary school teachers to investigate the correlation between teachers’ work-related quality of life and their organizational commitment [71]. The results showed a positive correlation between these variables. Nabati et al., piloted a study to examine the relationship between the quality of work-related life and organizational commitment among Iranian teachers and found a positive relationship [72]. Yildirim investigated the relationship between organizational commitment, occupational burnout, and self-efficacy among Turkish teachers [73]. The results showed a positive relationship between organizational commitment and self-efficacy. Lastly, Cheng and Gan found a positive relationship between occupational commitment and work-related quality of life among Chinese teachers [74].

Furthermore, moderate levels of teacher commitment, indicated by a score of 3.47 out of 6, reflect a balance between dedication and disengagement. Teachers with moderate commitment typically show reasonable involvement in their profession but might not feel deeply immersed in their roles. Their level of self-efficacy in their teaching abilities is likely moderate, meaning they feel capable but may not believe they can handle every classroom challenge. Similarly, job satisfaction at this level might reflect reasonable contentment with aspects of the teaching profession, such as relationships with students and autonomy, while leaving room for improvement in areas like administrative support or professional development.

Fresko et al., explored the impact of cultural differences on teacher commitment, highlighting how teachers in different cultural contexts exhibit varying levels of commitment [24]. In cultures that emphasize collective responsibility and communal goals, such as those found in Eastern societies, teachers tend to display higher levels of commitment than those in individualistic cultures, like Western countries, where personal achievement is more central. This variation in commitment levels can also influence self-efficacy and job satisfaction. A moderate level of commitment (M = 3.47/6) may be more representative of an individualistic context, where teachers might not feel fully integrated into a collective or supportive community, thus impacting both their sense of self-efficacy and overall job satisfaction.

Gaziel found a strong positive relationship between teacher commitment and job satisfaction. Teachers with higher levels of commitment reported greater job satisfaction [20]. In line with this, a moderate level of commitment (M = 3.47/6) would likely correlate with moderate job satisfaction, although it may not lead to the highest possible satisfaction. The strong correlation of r = 0.76 between self-efficacy and job satisfaction, as seen in Gaziel’s study, suggests that while moderate commitment might lead to moderate satisfaction, the overall satisfaction may still be somewhat limited compared to teachers with more substantial commitment or higher self-efficacy.

Tschannen-Moran and Hoy investigated the relationship between teacher self-efficacy and job satisfaction [35]. They found that higher self-efficacy significantly predicted teacher commitment and job satisfaction. For teachers with moderate commitment, their self-efficacy would likely be moderate, contributing to a more balanced but not exceptionally high level of job satisfaction. Given the strong correlation between self-efficacy and job satisfaction, moderate levels of self-efficacy would likely result in moderate job satisfaction, further reinforcing that teachers with moderate commitment might not experience the highest satisfaction levels.

In conclusion, the relationship between moderate teacher commitment, self-efficacy, and job satisfaction aligns with the findings of these studies. Moderate commitment tends to correlate with moderate levels of both self-efficacy and job satisfaction, with the correlation between self-efficacy and job satisfaction being particularly strong. While teachers with moderate commitment are likely content with certain aspects of their jobs, their overall satisfaction may not reach its peak unless they possess stronger self-efficacy and higher levels of commitment.

Overall, the results showed a positive relationship between teacher self-efficacy and quality of work-related life. The researchers attribute this to the intrinsic characteristics of teachers, such as motivation, perseverance, and passion for their work, which are among the main factors of the quality of work-related life that predict self-efficacy. These teachers continue in the profession with pleasure, enjoyment, and curiosity without considering external rewards and incentives. This also indicates their previous personal success, which raises their self-efficacy. Bandura noted that experiences of mastery are the primary source of success in nurturing a sense of self-efficacy [32]. Students’ respect and appreciation, and their positive attitudes toward the teacher, enhance the teacher’s social position. This represents social persuasion as one of the sources of self-efficacy, which reflects positively on teachers’ satisfaction with the quality of work-related life.

## Conclusion

This study demonstrates significant positive relationships between teacher commitment, self-efficacy, and work-related quality of life among Jordanian science teachers. The successful validation of the Arabic versions of the Teacher Commitment Scale, Teacher Self-Efficacy Scale, and Work-Related Quality of Life Scale offers reliable and culturally relevant instruments for future research in similar contexts. These scales provide an essential foundation for examining the complex interplay of factors contributing to teachers’ professional engagement and well-being.

Enhancing teacher commitment cannot be overstated, as it is a key determinant of school effectiveness and student learning outcomes. Previous research supports that a committed teacher is more likely to be motivated, engaged, and effective in the classroom [75]. By fostering an environment that supports teachers’ psychological well-being—such as promoting teacher self-efficacy and addressing factors that influence work-related quality of life—educational institutions can help create a more dedicated and effective workforce. This, in turn, contributes to improved educational outcomes.

However, while the positive correlation between teacher commitment and specific aspects of school functioning is clear, the direct impact of teacher commitment on overall school effectiveness remains a topic of ongoing debate. Studies from various international contexts have shown that teacher commitment can influence student outcomes indirectly through its effects on teaching quality, classroom environment, and teacher retention [35,52]. However, concrete evidence linking teacher commitment to direct student achievement remains inconclusive. More rigorous research is needed to explore how teacher commitment translates into tangible improvements in student performance and school effectiveness.

This study contributes to the growing body of evidence on the importance of teacher commitment and its impact on the educational system. It provides a basis for future investigations that could more definitively establish these relationships across educational settings.

### Recommendations

To address the challenges highlighted in the findings, specific evidence-based interventions should be offered to support teacher commitment and school effectiveness. One intervention involves implementing professional development (PD) programs to enhance teachers’ self-efficacy. Shaharabani and Tal emphasized that targeted PD initiatives can significantly strengthen teachers’ belief in their capabilities, fostering greater commitment to their roles [5]. More recent studies, such as Zhao et al., have also confirmed that self-efficacy–centered PD improves instructional practices and reduces teacher burnout, especially in rapidly changing educational environments [76].

In parallel, policies aimed at improving work conditions should also be prioritized. Royuela et al., demonstrated a clear link between quality of work life and job satisfaction, suggesting that better working environments contribute to more substantial organizational commitment [47]. This assertion is echoed in newer research by Alhija and Nir, which found that workload management, administrative support, and emotional well-being are critical determinants of teachers’ long-term engagement and retention. Implementing such policies can enhance individual teacher outcomes and promote broader school effectiveness [77].

### Limitations & future studies

While the study provides valuable insights into the relationships among teacher commitment, self-efficacy, and work-related quality of life, several limitations must be addressed in future research. One of the main limitations is the lack of direct evidence linking teacher commitment to student outcomes. While the study shows a positive association between teacher commitment and various aspects of school functioning, it does not establish concrete evidence on how teacher commitment translates directly into improved student performance or overall school effectiveness. This presents a gap in the research, as understanding the direct impact of teacher commitment on student outcomes is crucial for validating its importance in educational settings. Future studies should seek to explore this causal relationship more explicitly, using longitudinal data or experimental designs that could better capture the effects over time.

Another limitation is the generalizability of the findings, as the study is explicitly focused on Jordanian science teachers. While the results provide valuable insights into the Jordanian context, the cultural, social, and educational differences between countries mean these findings may not fully apply to teachers in other regions or educational systems. Teacher commitment and its related factors could vary significantly across different cultural settings, and as such, the results may need further validation in other contexts. Expanding the study to include teachers from various countries or educational environments would allow a more comprehensive understanding of how teacher commitment operates in diverse global contexts.

Additionally, if the study employed a cross-sectional design, it only captures data at a single point, providing a snapshot of the relationships among the variables. This design limits the ability to draw conclusions about causal relationships or to understand how these factors evolve. A longitudinal design would be more effective in tracking how teacher commitment, self-efficacy, and work-related quality of life develop over a more extended period and how these changes impact teacher performance and student outcomes in the long run.

The study potentially overlooks unmeasured variables influencing teacher commitment and work-related well-being. While it focuses on teacher commitment, self-efficacy, and work-related quality of life, other factors—such as school leadership, peer support, workload, and external stressors—could play significant roles in shaping teacher engagement and effectiveness. The study may present an incomplete picture of what influences teacher commitment by failing to account for these other variables. Future research should seek to control for these variables or explore them in addition to the primary factors studied.

Finally, while the study validates Arabic versions of the measurement scales, there may still be cultural and linguistic nuances that could impact how teachers interpret and respond to these instruments. Even though the scales were validated for Arabic speakers, there might be subtle cultural differences in how commitment, self-efficacy, and work-related well-being are understood across different regions or groups. This could lead to biases in how responses are collected, affecting the reliability and accuracy of the results. Future studies should continue refining and testing these instruments across diverse cultural and linguistic contexts to ensure they are universally applicable.

Addressing these limitations in future research would strengthen our understanding of teacher commitment and its impact on educational outcomes. This would provide a more comprehensive foundation for strategies to improve teacher well-being and student success.

Future studies can employ various research methods to understand better the factors influencing teacher commitment—such as organizational support, leadership styles, and cultural influences. Quantitative approaches, including surveys with validated scales and regression analysis, can help identify and measure the strength of relationships among these variables. Additionally, qualitative methods like interviews or focus groups can provide richer insights into how these factors are perceived and experienced by teachers in different contexts. Expanding research in this area can support the development of evidence-based interventions to enhance teacher retention and effectiveness, thereby contributing to the overall improvement of the educational system.

## Supporting information

S1 File Teacher Commitment Scale. (DOCX)

S2 File Teacher Self-Efficacy Scale. (DOCX)

S3 File Work-Related Quality of Life. (DOCX)

S1 Data Data-teacher. (SAV)
