# Correction: The relationship between teacher commitment, teacher self-efficacy, and work-related quality of life among science teachers

**DOI:** 10.1371/journal.pone.0334761

**Published:** 2025-10-14

**Authors:** Omar T. Bataineh, Ahmad M. Mahasneh, Zohair H. Al-Zoubi, Ahmad Qablan, Ahmed M. Alkaabi

There are errors in the author affiliations. The correct affiliations are as follows:

Omar T. Bataineh^1^, Ahmad M. Mahasneh^2^, Zohair H. Al-Zoubi^1^, Ahmad Qablan^3^, Ahmed M. Alkaabi^4^

1 Department of Educational Foundations and Administration, Faculty of Educational Sciences, The Hashemite University, Zarqa, Jordan, 2 Department of Educational Psychology, Faculty of Educational Sciences, The Hashemite University, Zarqa, Jordan, 3 Department of C&I, Faculty of Educational Sciences, The Hashemite University, Zarqa, Jordan and Department of C&I, College of Education, United Arab Emirates University, UAE, 4 Department of Foundations of Education, College of Education, United Arab Emirates University.
