# Supplementary material for: The relationship between teacher commitment, teacher self-efficacy, and work-related quality of life among science teachers
Source: PLoS One. 2025 Jul 1;20(7):e0326994. doi: 10.1371/journal.pone.0326994 (PMC12212536; doi:10.1371/journal.pone.0326994)
Supplement: S1 File — (DOCX) [file pone.0326994.s001.docx]

**Teacher Commitment Scale**

Dear Teacher,

The researchers are conducting a study titled "Professional Commitment of Teachers and Its Relationship with Their Self-Efficacy and Quality of Work Life." Kindly read the statements and mark an (x) in the appropriate place that reflects your opinion on each one, as these statements express thoughts, principles, and attitudes within a person’s character.

- Please answer all the statements with complete honesty and sincerity. Please note that this list is not a test, and there are no right or wrong answers.
- Please answer all the statements without exception. We appreciate your cooperation and thank you for your valuable contribution.
- Your answers are anonymous, so please do not write your name on this questionnaire.
- Please sign the consent form before taking the survey.

Thank you for your participation

| 1 | Often, I find it is difficult to agree with this organization’s policies on important matters relating to its employee. | *High Strongly Disagree* | *Strongly Disagree* | *Disagree* | *Agree* | *Strongly Agree* | *High*  *Strongly Agree* |
| --- | --- | --- | --- | --- | --- | --- | --- |
| 2 | I talk up this school to my friend as a great school to work for. |  |  |  |  |  |  |
| 3 | I find that my values and my school’s values are very similar. |  |  |  |  |  |  |
| 4 | All students can succeed and it is my mission to ensure their success. |  |  |  |  |  |  |
| 5 | It is my responsibility to ensure good social relations among my students. |  |  |  |  |  |  |
| 6 | I feel obliged to mediate among the rival groups of the students. |  |  |  |  |  |  |
| 7 | I used to be more ambitious about my work than I am now. |  |  |  |  |  |  |
| 8 | Sometimes I lie awake at night thinking ahead to the next day’s work. |  |  |  |  |  |  |
| 9 | I enjoy teaching. |  |  |  |  |  |  |
| 10 | If I could get a job different from being a teacher and paying the same amount, I would take it. |  |  |  |  |  |  |
| 11 | If I could do it all over again, I would not choose to work in the teaching profession. |  |  |  |  |  |  |
| 12 | I am disappointed that I ever entered the teaching profession. |  |  |  |  |  |  |
| 13 | The best decision that I have ever made was to become a teacher. |  |  |  |  |  |  |
