# Supplementary material for: The relationship between teacher commitment, teacher self-efficacy, and work-related quality of life among science teachers
Source: PLoS One. 2025 Jul 1;20(7):e0326994. doi: 10.1371/journal.pone.0326994 (PMC12212536; doi:10.1371/journal.pone.0326994)
Supplement: S2 File — (DOCX) [file pone.0326994.s002.docx]

**Teacher Self-Efficacy Scale**

(Schwarzer, Schmitz, & Daytner, 1999)

Dear Teacher,

The researchers are conducting a study titled "Professional Commitment of Teachers and Its Relationship with Their Self-Efficacy and Quality of Work Life." Kindly read the statements and mark an (x) in the appropriate place that reflects your opinion on each one, as these statements express thoughts, principles, and attitudes within a person’s character.

- Please answer all the statements with complete honesty and sincerity. Please note that this list is not a test, and there are no right or wrong answers.
- Please answer all the statements without exception. We appreciate your cooperation and thank you for your valuable contribution.
- Your answers are anonymous, so please do not write your name on this questionnaire.
- Please sign the consent form before taking the survey.

Thank you for your participation

|  | Item | Not At All True | Barely True | Moderately True | Exactly True |
| --- | --- | --- | --- | --- | --- |
| 1 | I am convinced that I am able to teach successfully all relevant subject content to even the most difficult students. |  |  |  |  |
| 2 | I know that I can maintain a positive relationship with parents, even when tensions arise. |  |  |  |  |
| 3 | When I try really hard, I am able to reach even the most difficult students. |  |  |  |  |
| 4 | I am convinced that, as time goes by, I will continue to become more and more capable of helping to address my students’ needs. |  |  |  |  |
| 5 | Even if I am disrupted while teaching, I am confident that I can maintain my composure and continue to teach well. |  |  |  |  |
| 6 | I am confident in my ability to be responsive to my students’ needs, even if I am having a bad day. |  |  |  |  |
| 7 | If I try hard enough, I know that I can exert a positive influence on both the personal and academic development of my students. |  |  |  |  |
| 8 | I am convinced that I can develop creative ways to cope with system constraints (such as budget cuts and other administrative problems) and continue to teach well. |  |  |  |  |
| 9 | I know that I can motivate my students to participate in innovative projects. |  |  |  |  |
| 10 | I know that I can carry out innovative projects, even when I am opposed by skeptical colleagues. |  |  |  |  |
